# Antifungal Drugs for the Treatment of Invasive Fungal Infections—A Limited Therapeutic Toolbox Facing Growing Resistances

**DOI:** 10.3390/ph18081220

**Published:** 2025-08-19

**Authors:** Victoria Susan, Mylène Lang, Marcela Sabou, Line Bourel-Bonnet

**Affiliations:** 1 Chemobiology and Pharmacognosy for Health (CPS) Team, Strasbourg Institute for Drug Discovery and Development (ITI IMS), Laboratory of Therapeutic Innovation (LIT), UMR 7200 CNRS/Unistra, Faculty of Pharmacy, 74, Route du Rhin, 67400 Illkirch, France; 2 University of Strasbourg Institute for Advanced Studies (USIAS), 67000 Strasbourg, France; 3 Strasbourg Institute of Parasitology and Tropical Pathology, Strasbourg University Hospital, 1–3 Rue Koeberlé, 67000 Strasbourg, France; amsabou@unistra.fr

**Keywords:** antifungals, invasive fungal infections, structure–activity relationships, resistance, drug candidates

## Abstract

Invasive fungal infections (IFIs) are one of the most significant public health challenges worldwide. Yet, research and communication thereof were left behind for a long time, until the WHO published a priority pathogens list to guide research, development, and public health action in October 2022. Indeed, due to the rising number of immunocompromised patients at risk and the high level of morbidity, mortality, and economic burden they entail, especially in low- and middle-income countries, IFIs are a serious public health threat. Fungal infections due to dimorphic fungi face additional challenges such as limited knowledge outside of endemic areas and restricted availability of antifungal molecules in areas affected by these infections. The number of related deaths per year is estimated at 2.5 million, but non-governmental organisations make a wider estimation, due to the difficulties in early in vitro diagnostic and troublesome collection and analysis of epidemiological data. Despite this fact, the therapeutic toolbox addressing these infections remains limited, with only four main families of molecules available so far. The antifungal therapeutic supply is composed of very toxic polyenes, the weakly selective and nearly unused 5-fluorocytosine, and azoles, some of which are becoming increasingly inefficient against IFIs. In the 2000–2020s, the fourth arising family consisted of safer semisynthetic echinocandins. Unfortunately, nowadays, more and more fungal isolates encountered in intensive care units exhibit a low susceptibility to echinocandins or are even multiresistant. In this review, we expose the current treatments available to fight against invasive fungal infections. We recall the discovery and physico-chemical aspects of these substances leading to structure/activity and structure/properties relationships. We particularly focus on the to-date resistances and their molecular mechanisms. We finally list some of the most relevant antifungal drug candidates, as they were freshly overviewed by the World Health Organization in April 2025, highlighting the importance of the molecular dimension of this pursuit toward the expansion of the antifungal therapeutic toolbox.

## 1. Introduction

Microbial agents have consistently interacted with human beings, playing a pivotal role in human evolutionary processes. The efficacy of this coexistence arises from their remarkable adaptability. Among microbial pathogens, fungi exhibit short generation times, flexible genomes, and a ceaseless ability to adapt to natural environments containing many adverse compounds, notably antifungal pesticides, thereby creating strong selection pressures [1]. However, fungal infections, especially invasive ones, are not sufficiently considered as a severe public health issue. Despite the growing interest of the scientific community for bacterial resistance to in-use antibacterial substances, the resistance of fungi to antifungal drugs remains underestimated and therefore under-reported [2]. Still, considering a potential 300 million severe cases globally resulting in an estimated 3.75 million fatalities annually [3,4], invasive fungal infections (IFIs) emerge as a significant public health challenge.

The fungal kingdom encompasses over six million distinct species [5], widely distributed throughout the natural environment. The overwhelming majority of microscopic fungi establish enduring colonies, existing within intricate communities in both environmental and human settings [6]. Nevertheless, fungi can also be primary or opportunistic pathogens, leading to fungal infections, also called ‘mycoses’. Firstly, superficial mycoses, including infections of the hair (ringworm), skin (athlete’s foot), or nails (onychomycosis), are more common in terms of overall global prevalence and are typically benign and manageable. On the other hand, invasive fungal diseases (IFDs) are associated with disproportionately high morbidity, mortality, and economic burden, especially for patients with weakened immune systems [4]. According to the European Organization for Research and Treatment of Cancer (EORTC) and the Mycoses Study Group Education and Research Consortium (MSGERC) criteria [7,8], a proven invasive or systemic fungal disease is confirmed when histopathological examination reveals tissue damages attributable to fungal elements and/or when the etiologic agent is isolated by culture from otherwise clinically sterile samples such as blood, tissues, or cerebrospinal fluid [9,10]. An IFD can be initiated via either invasion of fungal microbiota into mucosal surfaces, inhalation of fungal spores from the environment, or direct inoculation, leading to colonization, infection, and even dissemination [11]. To cause an IFD, the fungi must satisfy several criteria: (i) the capacity to grow at the human body temperature, (ii) the ability to invade internal tissues by penetrating or dodging host barriers, (iii) the capability to lyse tissues and absorb their components, and (iv) the aptitude to evade host immune defenses [12].

Because of their highly efficient and sophisticated immune system, humans are naturally resistant to IFDs. Therefore, one of the main factors that contributes to the rise in the emergence of IFDs is the increasing number of patients at risk. Among them, we can list (i) patients with a weakened immune system (aging, suffering from the flu, Acquired Immuno Deficiency Syndrom (AIDS), or uncontrolled diabetes, or presenting a treatment-induced immunosuppression, especially those facing onco-hematologic diseases), (ii) patients suffering from co-infections (*Mycobacterium tuberculosis*, cytomegalovirus, or HIV for the most frequently associated infecting agents), (iii) those undergoing invasive medical procedures (implantation of catheters or intravascular and intracranial devices, solid organ transplant, bone marrow graft), and (iv) those incurring traumas and deep burns [4,13]. The identification of new groups at-risk of IFDs is an ongoing process. One recent example involves COVID-19 and influenza pneumonia, which have led to a surge in the occurrence of comorbid invasive fungal infections, such as aspergillosis and mucormycosis [14,15,16]. For immunological aspects of this increasing risk, Kumar and coworkers recently published an instructive scheme of fungal cell wall interplay with host immune response during infections [17].

In late 2022 and for the very first time, the World Health Organization (WHO) published a fungal priority pathogen list presenting serious risks of mortality and morbidity (WHO FPPL) [18]. Thus, nineteen fungal species were classified into three priority groups (critical, high, and medium). The fungi that are the most often associated with IFDs (*Candida albicans*, *Candidozyma auris*, *Aspergillus fumigatus*, *Cryptococcus neoformans*, and Mucorales) are top-listed as critical [19]. Indeed, the rapid advent of resistance in usually susceptible pathogens and the emergence of novel multidrug-resistant fungal species (like the fearsome thermotolerant *Candida auris*, now called *Candidozyma auris* in the updated taxonomy) [20] are often associated with high mortality rates of IFDs and high hospitalization costs [2,21,22]. Drug-resistant fungi can rapidly spread in a variety of settings such as communities, healthcare facilities, and the environment including soil and water, causing IFDs in the above-listed at-risk patients. One can acknowledge this first official enlightening of such a global public health burden.

**Figure 1:**
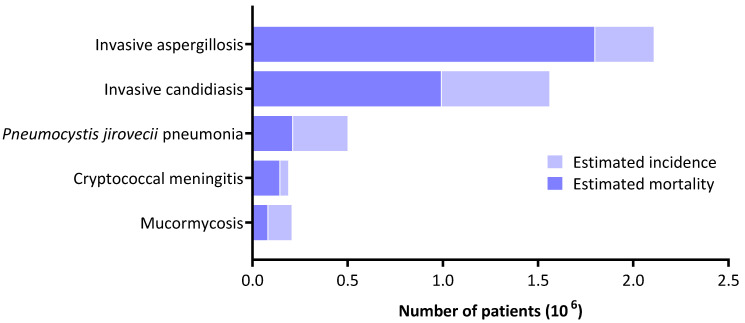
Global estimated incidence and mortality for the most common invasive fungal diseases affecting humans worldwide per year from 2019 to 2021 [4].

The global epidemiology of IFDs is difficult to assess because of the absence of national surveillance systems and the lack of available data in most countries. According to the Leading International Fungal Education (LIFE) initiative, the currently available estimations of the global incidence and mortality rates of IFDs are impacted by local healthcare programs, diagnostic tools, and the availability of antifungal drugs across the world [2,23]. Thus, there is a scarcity of epidemiological data accessible at present, and this paper only provides some examples of IFDs that are described in the literature (Figure 1). In addition, given the limited range of treatments and diagnostics available today for clinical use against IFDs, these are a significant concern for public health [4].

Invasive aspergillosis affects 2,113,000 humans worlwide per year, in the context of chronic obstructive pulmonary disease [13], intensive care [24], hematopoetic stem cell transplant [25], leukemia, and lymphoma [26]. Mortality rates may vary in these four population groups but are estimated at 85%, i.e., 1,801,000 deaths per year [4]. Invasive candidiasis is one of the most common opportunistic fungal infections, with an estimated 1,565,000 new cases worldwide per year, taking into account bloodstream *Candida* infections (candidemia) and invasive candidiasis without a positive blood culture (intra-abdominal abscess, peritoneal candidiasis, etc.) [4]. Mortality rates vary from relatively low numbers in less immunosuppressed patients to higher ones in those with more severe immunosuppression [27]. The mortality rates are estimated at 64%, i.e., 995,000 deaths per year [4]. *Pneumocystis jirovecii* currently stands as one of the leading microorganisms responsible for opportunistic infections in individuals with advanced AIDS. The worldwide prevalence of pneumocystosis is estimated to exceed 505,000 cases annually, and the mortality rates vary from 10 to 42% if the diagnosis is not delayed [4,28]. Cryptococcal meningitis remains common in uncontrolled AIDS cases [29], with about 194,000 new cases per year, and a 76% rate of mortality, i.e., 147,000 deaths. Mucormycosis is increasingly encountered in many settings, with an estimated 211,000 new cases a year, causing 84,000 deaths, corresponding to a 39% rate of mortality [4].

To treat IFDs, only four families of antifungal drugs are currently available. This figure contrasts with the more than ten families of antibacterial drugs a clinician can use to treat an invasive bacterial infection. Moreover, among every identified fungal pathogen, drug-resistant fungi account for over 50% of mortality rates attributed to IFDs [4,13]. In light of these challenges, some (too rare) efforts have been made to develop new antifungal drugs with original mechanisms of action. In contrast with the development of new antibacterial drugs, antifungal drug discovery and development is more challenging due to a somehow lesser scientific interest and, more importantly, the eukaryotic biochemistry. Consequently, many potential targets are similar to or also present in human cells, carrying a substantial host toxicity risk. As a result, currently available antifungal drugs have various drawbacks, notably in terms of safety and pharmacokinetic parameters. Thus, it is imperative to understand the limitations of clinically employed antifungal therapies. The mechanisms underpinning drug resistance are an additional preoccupation. Thus, it is highly recommended to consider different and complementary strategies to increase the number of new antifungal drugs that can address these problems. Therefore, one of the main challenges of antifungal drug discovery today is the exploration of new targets that are present exclusively in fungal cells (or to a lesser extent, mostly under-expressed in host cells).

**Figure 2:**
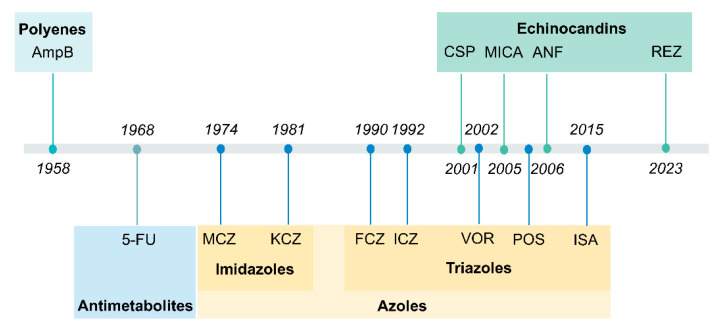
Timeline of antifungals used for treatment of invasive fungal diseases. The chronological points represent the years of FDA approval. AmpB: amphotericin B; 5-FU: 5-fluorocytosine (5-FC) as a precursor of cytotoxic 5-fluorouracil (5-FU); MCZ: miconazole; KCZ: ketoconazole; FLZ: fluconazole; ITZ: itraconazole; VOR: voriconazole; POS: posaconazole; ISA: isavuconazonium sulfate; CSP: caspofungin; MICA: micafungin; ANF: anidulafungin; REZ: rezafungin.

Until the end of the 20th century, mostly polyenes, antimetabolites, and azoles were routinely used to treat IFDs (Figure 2). These three families can cause serious side-effects and drug–drug interactions, which complicate their use in clinical settings. Oppositely, echinocandins, which are the most recent antifungal family, present milder side-effects [30]. A fifth family mainly composed of terbinafine is sometimes used, and antifungal combinations are clinically explored in last-chance protocols. Other antifungal drugs like allylamines, cyclopirox/cyclopiroxolamine and amorolfine are also available, but as they are mainly used for treating superficial dermatophytic infections, they are out of the scope of the present review.

## 2. Current Antifungal Substances Against Invasive Fungal Infections

### 2.1. Polyenes

Polyenes were the first class of antifungal agents to be discovered in the 1940s and deployed in clinical settings as soon as the 1950s [31]. Presently, only three polyenes (amphotericin B, nystatin, and natamycin) are in use [32], and only the first is employed against IFDs [33]. Polyenes were first identified as natural compounds isolated from the cultivation broths of *Streptomyces nodosum*, *Streptomyces noursei*, and *Streptomyces natalentis*, respectively [22,34]. In comparison to other antifungal agents, polyenes exhibit the broadest spectrum of activity. Both nystatin and natamycin possess high systemic toxicity levels, restricting their usage to topical applications only [32]. On the contrary, amphotericin B is the most prevalent polyene employed for systemic infections today [32]. Therefore, the timeline of IFDs treatments traces back to the approval of amphotericin B-deoxycholate in 1958 (Figure 2) [31,33,35].

**Figure 3:**
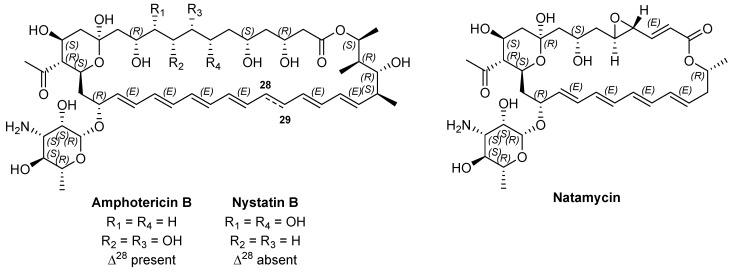
Chemical structure of clinically used polyene antifungals. Only amphotericin B is approved for the treatment of invasive fungal diseases. Nystatin and natamycin are used in topical applications only.

**Scheme 1:**
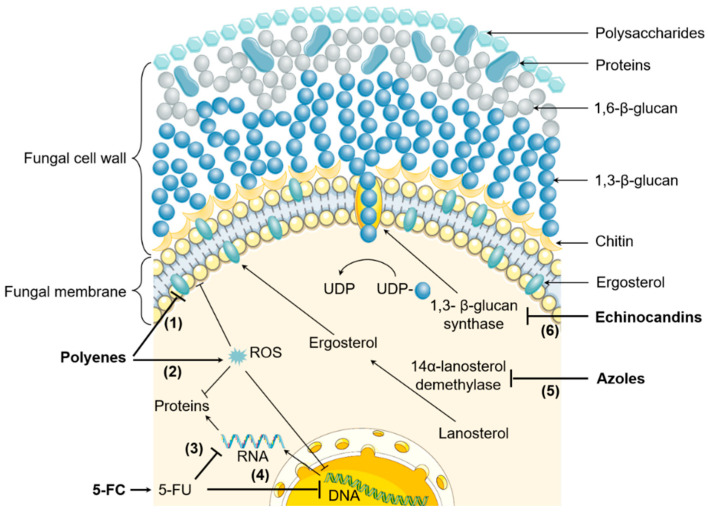
Representation of a yeast cell structure highlighting the main characteristics of the cell wall and membrane, as well as the targets of antifungal drugs used in the treatment of invasive fungal diseases. Polyenes bind irreversibly with ergosterol (1) and induce the formation of reactive oxygen species (ROS) (2). 5-Fluorocytosine is converted to cytotoxic 5-fluorouracil, which is incorporated into RNA (3), thereby inhibiting DNA synthesis (4). Azoles inhibit the key enzyme 14α-lanosterol demethylase involved in ergosterol biosynthesis (5). Echinocandins inhibit 1,3-β-D-glucan synthase, essential for fungal cell wall structure (6).

Amphotericin B is characterized by a polyunsaturated macrolactone ring linked with an extracyclic D-mycosamine saccharide (Figure 3) [30]. This amphiphilic structure facilitates the binding to the fungal membrane and an energetic interaction with ergosterol (Scheme 1) [36]. Current models emphasize membrane disruption via the “sponge” effect and pore formation as the primary mechanism of antifungal action, whereas reactive oxygen species (ROS)-mediated damage has lost prominence. These mechanisms have been recently reviewed by Bajaj et al. [37] and Cowen et al. [38]. Due to the structural proximity between ergosterol (predominantly found in fungi) and cholesterol (a constituent of human cell membranes), amphotericin B exhibits only ten-fold lower affinity for cholesterol, which explains its numerous side-effects and high toxicity [30]. The primary adverse effect of amphotericin B is nephrotoxicity, which arises from interactions with cholesterol-rich membrane cells, such as kidney cells [35,39]. The primary manifestations of nephrotoxicity include a decrease in glomerular filtration and loss of urinary concentrating ability [40]. Moreover, due to the leakage of monovalent ions (like Na^+^, K^+^, H^+^, and Cl^−^) consequent to membrane pore formation, polyenes can alter the ionic homeostasis [35].

The first marketed formulation of amphotericin B for the treatment of IFDs was a micellar dispersion which contains sodium deoxycholate as a solubilizer [35,41]. Despite its clinical efficacy against a wide range of IFDs, such as those caused by *Candida* spp., *Aspergillus* spp., and *Cryptococcus neoformans* [33,39], it can cause acute serious side-effects during intravenous administration, including fever, hypotension, dyspepsia, and nausea [41]. Furthermore, nephrotoxicity-related side-effects [35] significantly affect its clinical use. In the late 1970s, the research of drug delivery systems led to the development of a liposomal amphotericin B formulation [41]. There, amphotericin B is intercalated into the membranes of small unilamellar vesicles made from hydrogenated soy phosphatidylcholine, distearoyl phosphatidylglycerol, and, above all, cholesterol [41]. Due to the unique sterol/polyene affinity, amphotericin B is not released into the bloodstream, allowing for an improved tissue distribution [35,39,42]. Remarkably, those liposomes can interact with the ergosterol-rich fungal cell wall, facilitating the direct delivery of the drug to its fungal target [41]. Therefore, this formulation strategy considerably reduces infusion-related side-effects but does not completely eliminate the nephrotoxicity and hepatotoxicity problems [35,39]. Today, liposomal amphotericin B is employed as an empirical therapy for febrile or neutropenic patients and for treating IFDs in patients refractory to amphotericin B-deoxycholate or presenting a renal impairment [42]. It is recommended as a first-line treatment for central nervous system cryptococcosis and candidiasis (with or without flucytosine) [10], as a first-line treatment (at high doses) for mucormycosis [43], as an alternative to echinocandins in candidemia or ocular candidiasis [10], in azole-resistant aspergillosis or as combination therapy with azoles in treating invasive aspergillosis [44]. According to the WHO guidelines, it is also recommended for the treatment of histoplasmosis [45] or other endemic mycoses dues to dimorphic fungi [46] and cryptococcal meningitis [47] in patients infected by HIV. However, amphotericin B-deoxycholate is still commonly used today in low- and middle-income countries, mostly because of financial reasons [42].

### 2.2. Antimetabolites

**Figure 4:**
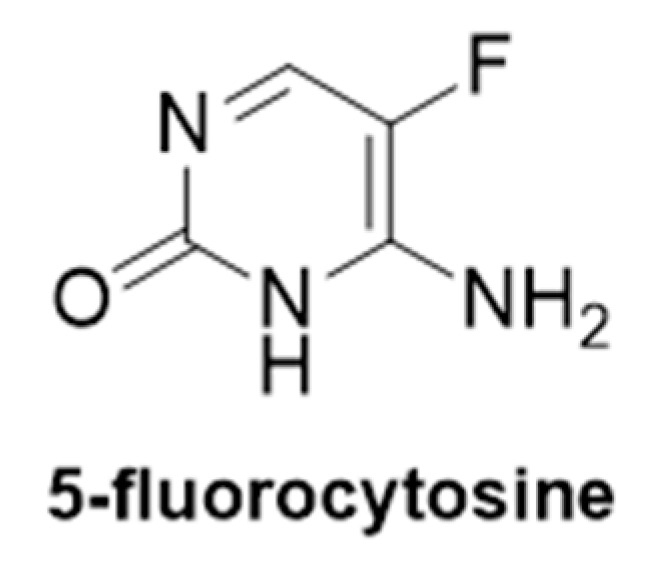
Chemical structure of clinically used antifungal antimetabolite 5-FC.

Today, the only clinically used antifungal antimetabolite drug is 5-fluorocytosine (5-FC, also called flucytosine). Here, we refer to the structural analogues of pyrimidine nucleic bases (Figure 4) that are essential for DNA, RNA, and protein synthesis, as well as metabolic reactions. 5-FC serves as a perfect illustration of drug repurposing within the pharmaceutical discovery process. Originally developed as a potential anticancer drug in 1957, 5-FC lacked efficient antineoplastic activity [48]. Ten years later, in 1968, this synthetic analogue of cytosine gained approval for use as an antifungal medication (Figure 2) [49,50,51]. Its mechanism of action was recently reviewed by Denning et al. [52]. It involves the active transport of 5-FC into fungi via membrane cytosine permease. Once inside the fungal cell, 5-FC is converted by cytosine deaminase to 5-fluorouracil (5-FU), which disrupts RNA and DNA synthesis (Scheme 1) [53]. Therefore, 5-FC can be regarded as a prodrug leading to an antimetabolite-based comprehensive activity. Interestingly, human cells lack cytosine deaminase, thus preventing the conversion of 5-FC to 5-FU. As a consequence, human cells are not directly affected by the toxic effects of the expressly formed 5-FU [53]. The major side-effects associated with 5-FC are primarily confined to bone marrow suppression [54]. Nevertheless, its use in monotherapy is now nonexistent owing to the rapid emergence of resistances, and 5-FC is rarely used in many countries [52].

### 2.3. Azoles

**Figure 5:**
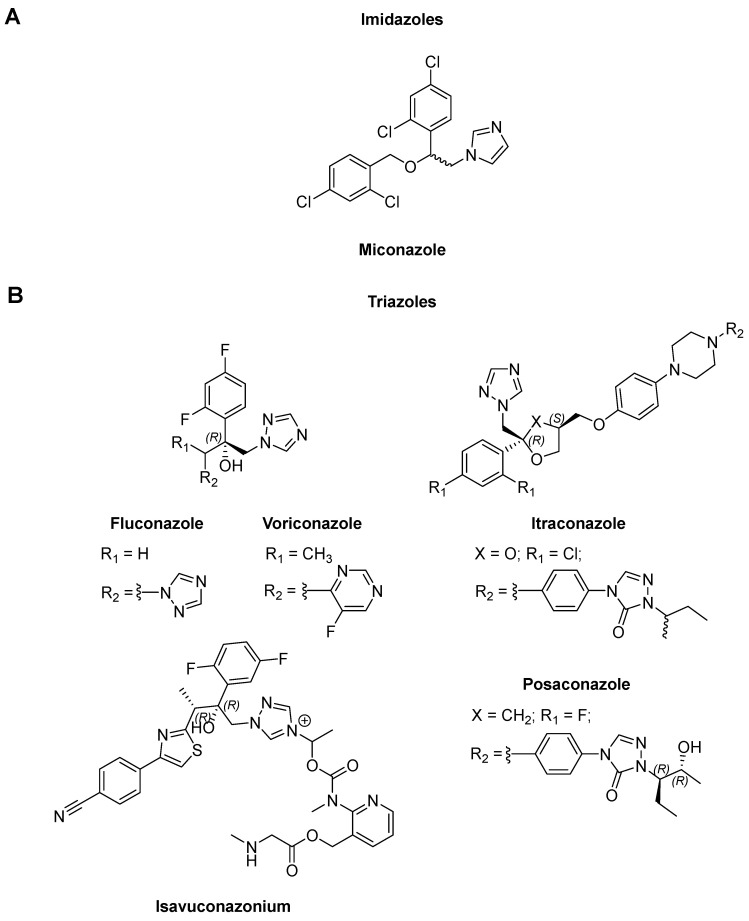
Chemical structure of clinically used azole antifungals for the treatment of invasive fungal diseases in Europe. Based on the number of nitrogen atoms in their heteroaromatic ring, they are classified in (**A**) imidazole and (**B**) triazoles.

Today, azoles are the most used antifungal drugs for the treatment of mycoses. These substances rapidly gained popularity due to their broad spectrum activity and oral availability [30]. Their pharmacophore is composed of a nitrogen-rich five-membered heteroaromatic ring attached to a quaternary carbon (Figure 5) [30]. Azoles can be classified into three groups based on the number of nitrogen atoms in their heteroaromatic rings: imidazoles, triazoles, and tetrazoles [55]. The discovery of antifungal azoles started from the serendipitous identification of antifungal properties of tritylimidazole [56]. The first marketed antifungal imidazole, chlorimidazole, was approved for topical use in 1958 [22]. However, it was not until 1974 and 1981 that the two imidazole-based antifungals, miconazole and ketoconazole, were approved for the treatment of IFDs (Figure 2) [30,32,57]. More recently, orally active ketoconazole was discarded from European guidelines for treatment of IFDs for hepatotoxicity reasons [58].

Subsequent to intensive research efforts in medicinal chemistry and synthetic screening, several triazole-based antifungals were approved for clinical use in IFDs from the 1990s to 2010s [30,31,34,57]. These include fluconazole, itraconazole, voriconazole, posaconazole, and isavuconazonium sulfate (Figure 2) [32,59]. Due to the emergence of resistances to azole agents [60,61], the bioisostere-based research of new antifungal agents yielded a new class known as tetrazoles.

All three sub-classes have a common mechanism of action consisting of the inhibition of 14α-lanosterol demethylase, which is crucial for the synthesis of ergosterol (Scheme 1). This binding via the azole nitrogen to the Fe^2+^-heme of the enzyme blocks demethylation, causing accumulation of aberrant methylated sterols that disrupt membrane fluidity and function [57]. Additional effects such as impaired morphogenetic transformation, reduced adherence, and direct membrane toxicity have been reported by Cowen et al. [62].

Because of the nonselective nature of the therapeutic target, azoles can cause side inhibitions of cytochrome-P450-dependent enzymes involved in human cell biosynthesis. This leads to high hepatotoxicity and numerous adverse effects, including hallucinations, hypokalemia, and QTc prolongation. Furthermore, the inhibition of cytochrome-P450-dependent enzymes and the strong binding of some azoles (for example, posaconazole) to plasmatic proteins can cause harmful drug–drug interactions with essential drugs, like atazanavir (antiretroviral), quinidine (antiarrhythmic), or erythromycin (antibacterial). Compared to imidazoles that were discovered and developed in the 1970s, triazoles that entered clinical practice from the 1990s to the 2010s have lower toxicity profiles [63]. This can be explained by a better affinity for fungal 14α-lanosterol demethylase than for human cytochrome-P450-dependent enzymes at therapeutic concentrations [57]. This is the major reason why imidazoles, excepting miconazole, are limited to the treatment of superficial mycoses [31]. In contrast, triazoles are used today for both superficial and systemic fungal infections [30]. Isavuconazole and voriconazole are first-line treatments for invasive aspergillosis [44], while fluconazole and voriconazole serve as alternatives to echinocandins for the management of candidemia [64]. Fluconazole is also used as consolidation therapy in cryptococcosis and in treating ocular candidiasis [10]. Itraconazole and voriconazole are recommended in infections due to dimorphic fungi [46], whereas isavuconazole and posaconazole are recommended in mucormycosis [65]. Despite their promising attributes, no tetrazole-based antifungal has been introduced for the treatment of IFDs so far.

### 2.4. Echinocandins

**Figure 6:**
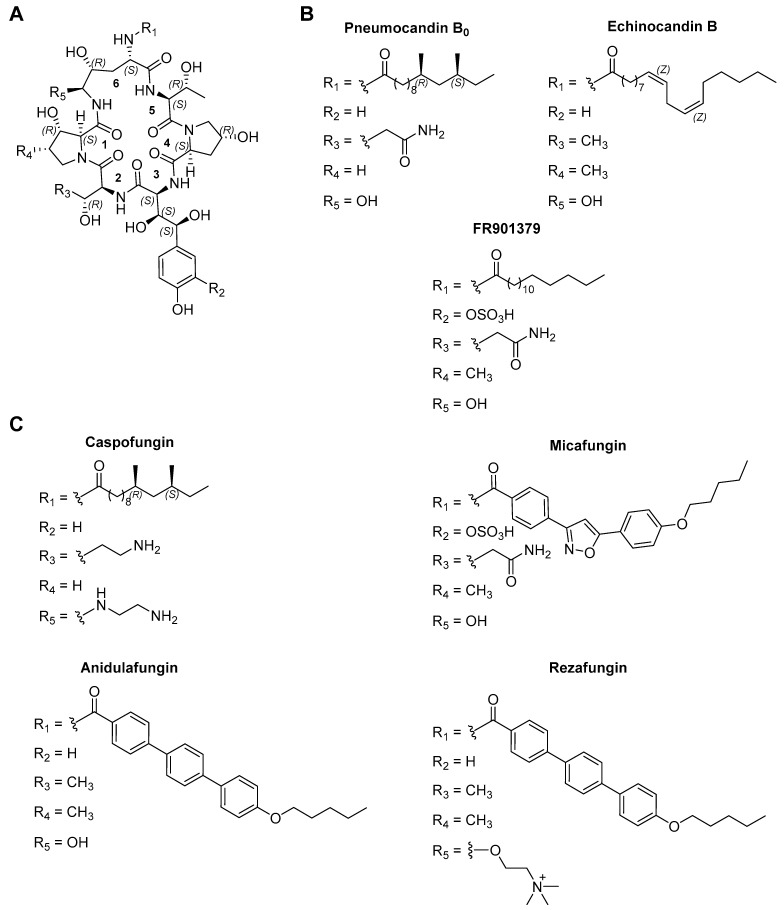
Chemical structure of echinocandins. (**A**) General structure of echinocandins. (**B**) Substituents of naturally produced precursors (pneumocandin B_0_, echinocandin B, and FR901379) and (**C**) clinically used echinocandins for treatment of invasive fungal diseases (caspofungin, micafungin, anidulafungin, and rezafungin).

Echinocandins are a particularly attractive class of antifungal drugs because of their original mechanism of action. This class currently comprises only four drugs available for clinical use: caspofungin, anidulafungin, micafungin [31], and the recent rezafungin (Figure 6) [30,66]. The eponymous member of this family, echinocandin B, was discovered in the 1970s in secretion broths of *Aspergillus nidulans* [67] and those of *Aspergillus rugulosus* [66,68,69]. Pneumocandin B_0_ was identified in 1987 as closely related to echinocandin B and produced by *Glarea lozoyensis* [70]. This culminated in the development of caspofungin, which was approved as the first echinocandin for clinical use in treating IFDs in 2001 [70,71]. Following this milestone, other echinocandin drugs were developed, including micafungin in 2005 [72] and anidulafungin, a derivative of echinocandin B, in 2006 [73]. Recently, the latest echinocandin, rezafungin, was approved by the Food and Drug Administration (FDA) in March 2023 as a once-per-week medication [74].

Echinocandins’ mechanism of action consists of the disruption of the fungal cell wall (Scheme 1). The latter is formed by several polymeric layers, including chitin over-layered by a matrix of 1,3-β-glucan and then 1,6-β-glucan (in yeast) or 1,3-α-glucan (in filamentous fungi). The architecture is completed by a final outer glycoprotein-rich layer. Echinocandins inhibit the membrane-bound protein 1,3-β-D-glucan synthase by binding non-competitively to the catalytic *FKS* sub-units [75]. This is a crucial enzyme in the biosynthesis of the 1,3-β-D-glucan polymer, which is present only in fungi [30]. Its function is to transfer a glucose sub-unit from a donor called Uridine DiPhosphate-glucose and attach it to the growing chain of glucan through 1,3-β-glycosidic linkage and to translocate the newly formed 1,3-β-D-glucan polymer into the extracellular space [76].

Typically, the structural stability of the fungal cell wall is largely dependent on the polysaccharide framework. The fungicidal activity against *Candida* spp. or fungistatic activity against *Aspergillus* spp. of echinocandins result from the alteration in the cell wall structure [77,78]. Interestingly, the echinocandin target is absent in human cells, which leads to lower toxicity profiles (especially lower hepato- and nephrotoxicity compared to other antifungal families) [79]. Therefore, echinocandins are now considered as a first-line therapy for invasive candidiasis and other forms of *Candida* infections (intra-abdominal abscess, peritonitis, endocarditis, and esophageal candidiasis) [10,71,73,74,79]. They are also used for the treatment of aspergillosis (with or without voriconazole) for patients who are refractory to other antifungal treatments or those who do not tolerate them [44,80,81,82]. Additionally, echinocandins are used for the prophylaxis of *Candida* infections in patients undergoing hematopoietic stem cell transplantation or patents with neutropenia [82]. However, echinocandins are complex compounds that emerged from evolutionary pressure between fungi without any consideration of cell membrane crossing, which is a critical point in pharmacokinetics. As lipopeptides, they have poor oral bioavailability and are consequently all used only by parenteral injection (over 1 h of infusion) [80].

Taking into consideration antimicrobial spectra, toxicities, and formulation issues, the current antifungal inventory is really restricted. This offers significant opportunities for the discovery and development of new drugs. Unfortunately, the current pipeline is lightly filled, as was underscored by the WHO in April 2025 [83] in a second striking publication.

## 3. Spectrum of Activity of Antifungal Substances and Resistances Thereto

The antimicrobial spectrum of activity refers to the range of fungal species an antimicrobial agent can effectively inhibit. To evaluate the sensitivity of a pathogen to an antifungal agent, in vitro susceptibility studies are performed. The latter enable the quantification of either minimal inhibitory concentration (MIC) or minimal effective concentration (MEC) values. For example, MIC_50_ and MIC_90_ are usually used as the minimum concentrations of an antifungal agent that inhibit the growth of 50% and 90% of the isolates of the tested fungal population, respectively, relative to a growth control. It is usually given in a microgram per milliliter (µg.mL^−1^) unit, whereas a result in micromole per milliliter (µmol.mL^−1^) would be preferable to reliably compare compounds whose structures (and molecular weights, consequently) are frankly different. On the other hand, the MEC is defined as the minimum concentration of an antifungal that causes the growth of aberrant short hyphal segments. The European Committee for Antimicrobial Susceptibility Testing (EUCAST) guidelines [84] and Clinical and Laboratory Standards Institute (CLSI) [85] have developed broth microdilution protocols that are used today as standardized methods to assess these values. Both EUCAST and CLSI protocols include the use of defined concentrations of antifungal agents in liquid culture media [84,85]. To determine the MIC of an antifungal substance, both organizations have similar criteria. For flucytosine, azoles, and echinocandins, the 50% inhibition is determined in both protocols. For amphotericin B, complete growth inhibition is required by CLSI, whereas only a 90% growth decrease is required by EUCAST. However, important differences between these two methods may significantly impact the MIC determination [86]. Notably, the preparation of the putative antifungal substances, the inoculum size, and the glucose content in the culture media are different [86]. Also, in the CLSI method, the MIC determination relies on visual reading using a viewing mirror [85], whereas the EUCAST method employs a spectrophotometer microplate reader [84], providing more reliable and repeatable results. Alternative methods for assessing MIC values, such as disk diffusion, present a high inter-laboratory variability [86]. This explains the variability in MIC and MEC values reported in the literature.

As versatile and potent microorganisms, fungi can develop mechanisms to withstand unfavorable environmental conditions. This adaptation capability is a key reason why fungal species that are normally susceptible to an antifungal agent can acquire resistance to it. Moreover, fungi can be multinucleated and/or multicellular and can carry multiple chromosomes, providing enhanced opportunities for genetic modifications and emergence of resistances due to selection pressures from repeated exposure to an antifungal agent (Table 1) [60]. Phenotypic heterogeneity can also impact antifungal susceptibility. Like bacteria, fungi can form organized and complex communities called biofilms that consist of fungal cells surrounded by an extracellular matrix mainly composed of polysaccharides [30]. This acts as a barrier that sequesters antifungal drugs, preventing their access to the fungal cells [21,87]. Therefore, biofilm formation is a non-genetic strategy developed by fungi to resist to antifungal drugs and to survive [88].

### 3.1. Resistances to Polyenes

Polyenes are a class of antifungals with fungicidal properties in most fungi. Resistance to this antifungal family is uncommon, but it can be intrinsic for fungi such as *C. haemulonii* [93], *C. auris* [33], *C. lusitaniae* [33], *Trichosporon* spp. [94], *A. terreus* and *A. flavus* [95], *Scedosporium* spp. [33], *Lomentospora* spp. [33], and *Fusarium* spp. [33]. The fact that resistance to polyenes is a rare occurrence might be the consequence of the severe fitness trade-off related to its acquisition [62] and to the fact that polyenes target a major wall component, not just an enzyme, which other antifungal families target [33].

Amphotericin B exerts its functions at the fungal plasma membrane and extracellularly, which determines mechanisms of resistance different from the antifungals with an intracellular activity. Ergosterol is the target of polyenes, and the proportion of ergosterol and alterations in the membrane sterol composition can affect susceptibility to them. A reduction in ergosterol content can be a consequence of mutations in genes like *Erg1*, *Erg2*, *Erg3*, *Erg4*, *Erg5*, *Erg6*, *Erg11*, and *Erg13*, which can lead to a lower level of the therapeutic target [33,93].

Polyenes also promote ROS formation and their accumulation in fungal cells, which leads to oxidative stress. Yet, the degree of this accretion can vary from species to species and from isolate to isolate. *Candida haemulonii*, a yeast species showing a high proportion of amphotericin-B-resistant isolates, is able to tolerate a higher concentration of ROS compared to other *Candida* species [93]. Heat-shock proteins 90 (HSP90) and 70 (HSP70) are key players in controlling cellular stress, and genes encoding for these proteins have been associated with amphotericin B resistance in *A. terreus* [33], *C. auris* [96], and *Trichosporon* spp. [93]. HSP90 stabilizes signal transductors implicated in antifungal-induced stress in *Candida*, *Cryptococcus*, and *Aspergillus* species, but their roles in the acquisition and maintaining of polyene resistance need further investigation [62].

Another mechanism of resistance to amphotericin B described in *C. haemulonii* is the shift from aerobic respiration to fermentation [93]. This phenomenon is induced by amphotericin B and leads to a decrease in intracellular ROS. In addition to altered metabolic status, iron homeostasis and post-translational modification are other mechanisms implicated in amphotericin B resistance [96].

### 3.2. Resistances to 5-FC

Flucytosine disrupts nucleic acid biosynthesis within the cell, and it requires activation within the fungal cell via metabolism by the pyrimidine salvage pathway [89]. It is active against *Cryptococcus* spp., most *Candida* spp., and some agents of chromoblastomycosis, and it has limited effect on molds [88]. Flucytosine resistance is rapid and extremely frequent, which discards monotherapy. This can be attributed to a defect in intracellular penetration or to a shortcoming of the metabolism of the prodrug into 5-FU, which is the active cytotoxic agent [88,90]. In pathogenic *Candida* spp., loss or mutations in *Fca1/Fcy1*, *Fcy2*, *Fur1*, and *Msh2* genes have been implicated in resistance to 5-FC [89]. Its place in antifungal treatment is mostly against cryptococcal meningitis as part of combinations with amphotericin B—shown to accelerate the rate of clearance of the infective agent and reduce mortality, and with fluconazole—demonstrated to prevent the selection of resistant strains [1]. Flucytosine associated with amphotericin B is also used in treating *Candida* endocarditis, endophtalmitis, and hepatosplenic or osteoarticular infections [50].

### 3.3. Resistances to Azoles

Azoles target the fungal cell membrane and block ergosterol biosynthesis, leading to intracellular build-up of a toxic sterol. They have a fungistatic effect on yeasts and fungicidal activity on *Aspergillus* spp. [62]. *Pichia kudriavzevii/Candida krusei* is innately resistant to fluconazole, and 87–100% of *C. auris* are reported as resistant to these antifungals [89,97]. *C. glabrata’s*, now called *Nakaseomyces glabratus*, azole resistance varies from 9–10% in the SENTRY Antifungal Surveillance Program [98] to 86% in Slovenia and 100% in Croatia, as reported by a multinational study looking into candidemia trends [99]. The same authors found 73% of *C. parapsilosis* strains to be resistant to azoles in Italy and 81% in Croatia [99]. Fluconazole is not active on molds, and *Aspergillus* can become resistant to the other triazoles, with reported data varying from 0.3% in Austria to 13% in the UK and 25% in the Netherlands [1,95]. Heteroresistance is defined as the presence of intrinsic resistance to a drug in a minority subpopulation that could be selected by treatment and become dominant. This has been described for fluconazole with *C. neoformans* and *C. glabrata* [1].

Many mechanisms can be responsible for azole resistance and can even be combined in a single isolated resistant strain. Among the main mechanisms one can find (i) the modification of 14α-demethylase by mutation of the corresponding encoding gene; (ii) the overproduction of the target; (iii) the overexpression of efflux pumps leading to a collapsed bioavailability of the azoles and (iv) compensating modifications of other steps of ergosterol biosynthesis leading to the overcoming of the target enzyme inhibition.

Drug target alteration or overexpression of the *Erg11* gene in yeasts and *Cyp51* in molds is a well-explored and common mechanism leading to acquired azole resistance. In the case of *C. albicans*, 140 different *Erg11* amino acid substitutions have been described, the majority within three hot-spot regions [62,89]. In *A. fumigatus*, the Cyp51 isoenzymes are encoded by genes *Cyp51 A* and *Cyp51 B*, and while the former has been well-studied and characterized, the role of the latter is less understood [62]. Intrinsic azole resistance in *A. lentulus* has also been linked to the *CypA* gene [95]. Specific amino acid substitutions like TR34/L98H and TR46/Y121F/T289A coupled with duplications in the *Cyp51A* promoter gene are associated with azole resistance in environmental and clinical isolates [95]. Mutations in the *Erg3* gene have been shown to determine azole resistance in *C. albicans* and *C. parapsilosis* [1].

Resistance to azoles also involves upregulation of plasma membrane efflux pumps responsible for reduced accumulation of these intracellular drugs [62]. Higher efflux pump activity has been reported in the *C. haemulonii* species complex, which has low susceptibility to azoles and echinocandins [93]. *Candida* biofilms have intrinsic antifungal resistance, and in some cases, the fluconazole concentration required for inhibition is a thousand times higher when compared to planktonic cells [89].

### 3.4. Resistances to Echinocandines

Echinocandins target the fungal cell wall via inhibition of (1,3)-β-D-glucan synthase. Mucorales and basidiomycetes such as *C. neoformans* are intrinsically resistant, but the members of this antifungal family are fungicidal on *Candida* and fungistatic on *Aspergillus*.^65^ Out of the less susceptible yeast species, 2–13% of *C. glabrata* and 0–8% of *C. auris* have been reported as resistant to echinocandins [1,89]. *C. parapsilosis*, *Meyerozyma/Candida guilliermondii*, and *C. haemulonii* species complex also have low susceptibility to this drug class [89,93]. Attenuated antifungal activity at high drug concentrations is known as the paradoxical growth effect or “Eagle effect” and characterizes caspofungin and molds, mostly *Aspergillus* [46].

It is well-established that mutations in the *Fks* genes (*Fks1*, *Fks2*, and *Fks3*), coding for the catalytic sub-unit of 1,3-β-D-glucan synthase, results in reduced susceptibility to echinocandins [100]. In fact, mutations such as point mutations, gene duplications, and transposon insertions are common in fungi [70]. Thus, mutations of *Fks* genes are found in highly conserved regions called hot-spots [62]. The majority of mutations described in *Candida*, *Aspergillus*, and *Cryptococcus* isolates presenting an acquired resistance to echinocandin hot-spots are present in the *Fks1* gene, which is an essential gene in *C. albicans*. In contrast, in *C. glabrata*, *Fks1* and *Fks2* are functionally redundant for viability, and mutations can also occur in the *Fks2* gene. In *Aspergillus*, *Fks1* mutations in vivo are associated with a high fitness cost and resistance in rare cases [62].

The acquisition of resistance to echinocandins is strongly dependent of the position of and specific amino acid mutations, which can be substitutions, deletions, or insertions. These mutations reduce the catalytic efficiency of 1,3-β-D-glucan biosynthesis, inducing modifications in the cell wall composition [62]. Moreover, *Fks* gene mutations can cause cross-resistance to all echinocandins [101]. In *C. glabrata* and *C. parapsilosis*, mutations in the *Erg3* gene can also be acquired upon echinocandin exposure in vitro and result in resistant strains and determine cross-resistance to fluconazole [1].

Fungi also present compensatory adaptive mechanisms that can induce cell wall repair in response to disruption by echinocandins. The cell stress induced by the inhibition of β-glucan synthase indirectly activates the Protein Kinase C–Mitogen-Activated Protein Kinase (PKC-MAPK) pathway, which is responsible for intermittent reconstruction of the cell wall, through the upregulation of chitin and mannan [101]. The compensatory chitin synthesis can also be activated by the Ca^2+^/calcineurin and high-osmolarity glycerol (HOG) pathways [62,91], which are involved in drug tolerance. Remarkably, *Fks2* expression in *C. glabrata* is calcineurin-dependent, suggesting that it could be regulated synergistically with calcineurin inhibitors [92,102]. The upregulation of chitin has also been shown to be calcineurin-related in *C. albicans* and *A. fumigatus* [1].

There is a growing interest in the implication of epigenetic modifications in antifungal acquired resistance, but their impact needs to be explored further.

## 4. Novel Antifungal Substances in Development

Due to multiple gaps in the current antifungal arsenal, including spectrum, toxicity, and formulation issues, increasing opportunities for new drug development against invasive fungal infections have arisen. Today, the antifungal mode of action and resistance is better understood thanks to chemical biology improvements and live-cell imaging. Thus, relevant fluorescent molecular tools based on the scaffold of popular antifungals have enhanced the study of their effects on fungi at the subcellular level [103].

To overcome the above-mentioned limitations, scientific studies have focused on some strategies such as (i) chemical derivatization of current antifungal families, (ii) design and synthesis of original scaffolds toward new first-in-class antifungals acting on relevant targets, and (iii) discovery and development of natural and semisynthetic products with potential antifungal activity. Other modern strategies, including (iv) dual-targeted therapy and combination, as well as (v) repurposing and label extension, are also very promising. Here, we briefly describe representative drug candidates and their perspectives. For a comprehensive up-to-date overview of the drug candidates, the reader is advised to consult the WHO freshly published report entitled *Antifungal agents in clinical and preclinical development* [83] and the linked *Antifungal preclinical and clinical pipeline review* that can be freely downloaded.

**Figure 7:**
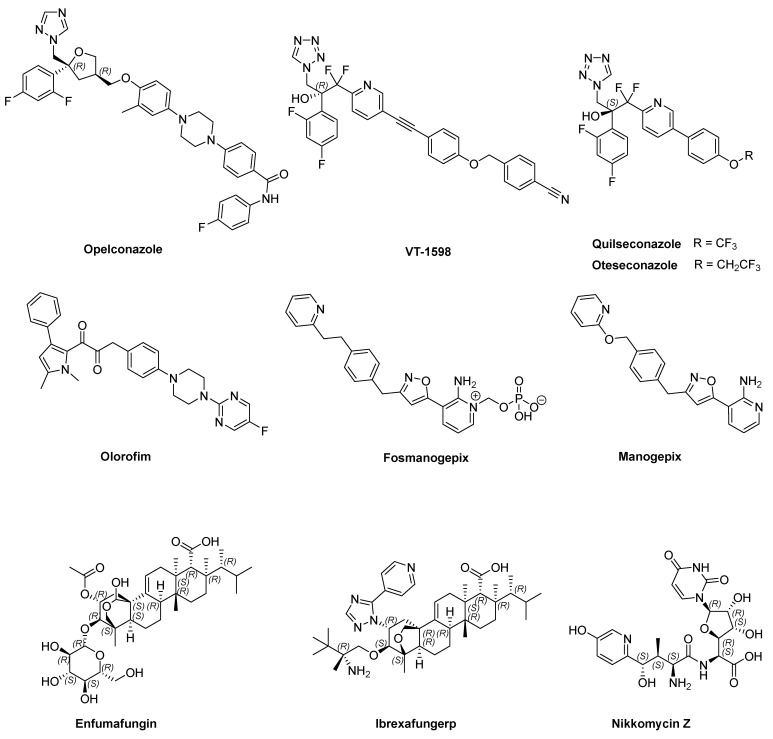
Chemical structures of antifungals in development or reconsidered against IFIs.

**Scheme 2:**
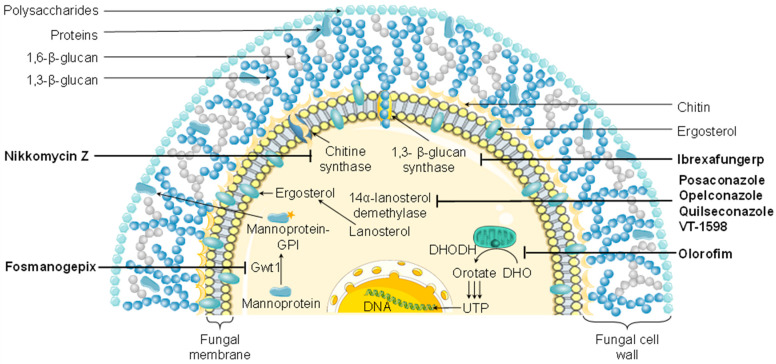
Schematic representation of the mechanism of action of potential antifungal agents under development for the treatment of invasive fungal infections. The new azoles—posaconazole, opelconazole, quilseconazole, and VT-1598—act by inhibiting the key enzyme in ergosterol synthesis, 14α-lanosterol demethylase, thus disrupting cell membrane structure. Olorofim targets dihydroorotate dehydrogenase (DHODH), an enzyme responsible for the transformation of dihydroorotate into orotate, which is involved in the biosynthesis of pyrimidines necessary for nucleic acid synthesis. Ibrexafungerp inhibits the key enzyme in the biosynthesis of 1,3-β-glucan, 1,3-β-glucan synthase, like echinocandins, which impacts the structure of the fungal cell wall. Fosmanogepix inhibits the Gwt1 enzyme, which is essential for the establishment of the glycosylphosphatidylinositol (GPI) label, enabling transfer of mannoproteins to the fungal cell wall. Nikkomycin Z blocks chitin synthase, which is responsible for chitin synthesis in the fungal cell wall.

(i) Chemical derivatization is extensively used in antifungal drug discovery and consists of synthesis and development of new derivatives, aiming at reducing toxicity, minimizing drug–drug interactions, and identifying new drug candidates to overcome the emerging resistances. Therefore, this strategy led to the discovery of new promising azoles, such as opelconazole, oteseconazole, quilseconazole, and VT-1598 (Figure 7 and Scheme 2). Opelconazole is a long-acting triazole designed for inhalation using nebulized delivery to treat invasive pulmonary fungal infections, while limiting drug absorption into the systemic circulation, systemic toxicity, and drug–drug interactions [1,104]. This new azole is active against a wide range of fungi, including *C. albicans*, *C. glabrata*, *C. krusei*, *A. fumigatus*, and *C. neoformans* [105,106]. In contrast, *A. niger* and *Fusarium* spp. present an intrinsic resistance to it [105]. Opelconazole is currently being studied in phase 3 trials for the treatment of refractory invasive pulmonary aspergillosis in combination with other systemic antifungal therapies (NCT05238116; NCT05037851) [106]. Therefore, inhaled opelconazole may be an important therapeutic progress for these patients, given the limited number of local effective drugs. Another notable advance in the azoles antifungal family is the development of tetrazoles, which have fewer side-effects and fewer drug–drug interactions compared to triazoles [1,54].

Three tetrazole compounds that have been or are currently under investigation include the following: (a) VT-1161 (oteseconazole), approved by the FDA in April 2022 but rejected by the EMA in August 2023 due to concerns regarding both quality and efficacy. Currently, no clinical trials are registered for this compound; (b) VT-1129 (quilseconazole), which was granted Fast Track designation by the FDA in June 2016 for the treatment of cryptococcal meningitis; and (c) VT-1598, currently under clinical evaluation (NCT04208321), which has shown promise in treating invasive fungal diseases (IFDs) caused by a broad range of pathogens, including *C. auris* [107], *Aspergillus* spp. [107], *Cryptococcus* spp. [108], and *Coccidioides* spp. [109].

(ii) Exploring novel pathways and cellular components is crucial for developing new antifungal agents. In vitro screening of chemical libraries against *A. fumigatus* led to the discovery of olorofim (Figure 7), which targets dihydroorotate dehydrogenase (DHODH) [110]. Due to its good oral bioavailability, olorofim can be administered both orally and intravenously [111]. Olorofim disrupts pyrimidine biosynthesis, affecting crucial cellular processes for fungal survival (Scheme 2). This small molecule presents a wide antifungal activity against molds, including *Aspergillus* spp., *Scedosporium* spp., and dimorphic fungi such as *Histoplasma* spp. and *Coccidioides* spp. [105,111]. In contrast, olorofim is inactive against *Candida* spp., Mucorales, and *C. neoformans* [1]. Ongoing clinical trials (NCT05101187) are evaluating its efficacy for treating invasive fungal infections and precisely invasive aspergillosis, potentially addressing azole-resistant strains [112,113].

Another first-in-class antifungal agent developed in recent decades is fosmanogepix (APX001), the prodrug of manogepix (Figure 7) [1]. Fosmanogepix has high oral bioavailability and can be administered orally or intravenously [105]. Studies on the biosynthesis of fungal cell wall mannoproteins led to the identification of 1-(4-butylbenzyl)isoquinoline, which, after optimization, culminated in the development of manogepix [114]. Fosmanogepix inhibits the GPI-anchored wall transfer protein 1 (GWT1) enzyme, disrupting fungal cell wall integrity, reducing hyphal growth and virulence (Scheme 2) [114]. In vitro studies of manogepix revealed an antifungal activity against yeasts including *C. auris*, *Cryptococcus* spp., and *Coccidioides* spp. [114]. Moreover, it also showed fungistatic activity against molds such as *A. fumigatus*, *Scedosporium* spp., *Fusarium* spp., and *L. prolificans* [114,115]. Phase 2 clinical trials showed promising results for treating candidemia and other forms of invasive candidiasis, and fosmanogepix is currently in phase 3 trials (NCT05421858) [116,117]. Remarkably, fosmanogepix has high oral bioavailability and can be used either orally or intravenously [115].

(iii) Natural products have historically been essential in drug discovery, with nature-originating antifungal substances evolving as survival mechanisms. An example of recently discovered antifungal natural products is enfumafungin, identified through high-throughput screening and produced by endophytic fungal species [22]. The optimization of the molecule led to the development of ibrexafungerp (Figure 7), a semisynthetic antifungal targeting, as also targeted by echinocandins, 1,3-β-D-glucan synthase (Scheme 2) [30]. Unlike echinocandins, ibrexafungerp binds to a different site of the fungal enzyme and is orally bioavailable [1], showing potent activity against echinocandin-resistant *Candida* species, such as *C. glabrata* and *C. auris* [118]. In vitro studies have also shown antifungal activity against *Cryptococcus* spp., *Aspergillus* spp., *Histoplasma* spp., *Coccidioides* spp., and *Blastomyces* spp. [105,118]. Despite its approval for vulvovaginal candidiasis in June 2021 [119], ibrexafungerp is not yet available for invasive fungal infections. Pending results from recent phase 3 trials (NCT03059992) [120] may soon expand its indication. Another interesting example of antifungal natural products is nikkomycin Z, initially isolated from *Streptomyces tendae* [121]. This first-in-class antifungal is a peptidyl nucleoside that inhibits chitin synthase (Scheme 2) and shows antifungal activity against *Coccidioides* spp. by disrupting fungal cell wall integrity [122]. Phase 1 trials are completed, but phase 2 trials for treatment of coccidioidomycosis were terminated due to funding and recruitment issues [123]. Nonetheless, continued exploration and design of nikkomycin analogs remain promising.

A recent publication has listed Fungal Kinase 1 (FK1) modulators that influence β-1,3-glucan synthesis in fungi. Among them, we find some preclinical candidates of a diverse chemical nature: (i) lipocyclopeptides like mulundocandin, aculacin A, acrophiarin, and arborcandin; (ii) cyclopeptides like clavariopsin; (iii) glyco-triterpenes like enumafungin and ascosteroide; (iv) triterpenes like ergokonin C; and (v) other scaffolds like chaetiacandin, fusacandin, papulacandin, and corynecandin [17]. Such a list is very encouraging and supplies the pipeline with interesting preclinical candidates to combat fungal infections.

(iv) In research nowadays, dual-targeted therapy against fungi is a promising strategy to combat drug-resistant fungal infections and improve treatment efficacy. Among the related key approaches, first, one can find membrane and DNA targeting, showing potent antifungal activity against multidrug-resistant strains such as *Candida* spp., *A. fumigatus*, and *Cryptococcus*. This approach prevents the development of resistances and enhances selectivity compared to antifungals like amphotericin B [124]. Another dual therapy is efflux pump inhibition combined with Target Of Ripamycin Complex 1 (TORC1) signaling, which is achieved by beauvericin, a natural product, to treat resistant fungal infections. It also activates protein kinase Casein Kinase 2 (CK2) and inhibits HSP90, disrupting fungal resistance mechanisms [125]. A third reported strategy is DNA binding in fungal nuclei. There, antifungal luminogens penetrate fungal membranes, depolarize them, and bind to DNA, leading to DNA damage. These compounds have shown efficacy in vitro and in murine models of *C. albicans*-induced vaginitis [126]. An additional double target is ergosterol biosynthesis coupled with immune activation. In this strategy, novel bifunctional inhibitors block ergosterol biosynthesis while inducing ROS production and fungal apoptosis. Additionally, these compounds enhance immune activation against fungal infections [127]. To illustrate the host–pathogen pathway targeting, dual inhibitors targeting sphingosine-1-phosphate lyase (SPL) in both fungi (*A. fumigatus*) and humans modulate immune responses while reducing fungal fitness. This paves the way for therapies that benefit the host while combating infections [128]. In parallel, as mentioned above, combining antifungal agents with different mechanisms of action (e.g., voriconazole with caspofungin or liposomal amphotericin B) has shown synergistic clinical effects in reducing fungal burden and improving survival rates in invasive fungal infection [129,130], and a synergy is expected in the combinations that will be tried in the future.

(v) Finally, a repurposing strategy has been undertaken for substances like antibacterial clindamycin, antidiabetic pioglitazone, antidepressant sertraline, and anticancer tamoxifen [83]. Label extension is carefully studied for caspofungin, fosravuconazole, ibrexafungerp, isavuconazolium sulfate, rezafungin, polymer-based super-bioavailable (SUBA) itraconazole, and voriconazole [83].

## 5. Conclusions

Invasive fungal infections are a serious health threat but are not sufficiently taken into account in spite of millions of patients being at risk. Only four families of antifungal substances are commonly used in therapeutics to fight against IFIs, limited to mainly three if we consider 5-FC’s restricted use. As bacteria achieve against antibacterial drugs, fungi develop several mechanisms to resist antifungals. The emergence of resistance to existing antifungal agents, coupled with the limited antifungal armamentarium, underscores the critical need for the discovery and development of new antifungal modalities.

This review emphasizes past and current advances in antifungal drug discovery. It also lists the multiple mechanisms by which fungi overcome antifungal action. There is an urgency of exploring new targets and original mechanisms of action to identify unprecedented antifungal agents. Booming structural data and increasing bioproduction skills together with purposely designed fluorescent molecular tools and ever-sophisticated live-cell imaging pave the way to unprecedented knowledge. In this context, the medicinal chemistry tools, through structural modifications and molecular diversity pursuit, play a significant role in the development of new, effective, safer, and more affordable antifungals. Advantageously, dual-targeted therapy and drug combinations enhance efficacy against drug-resistant pathogens and reduce the likelihood of resistance development. This multifaceted approach holds great promise for addressing the rising challenge of antifungal resistance and advancing therapeutic options for invasive fungal diseases. A comprehensive interdisciplinary approach is essential for safeguarding public health and advancing the field of antifungal therapeutics. In this perpetual fight, the October 2022 WHO publication of the *Fungal Pathogen Priority List* and the recent April 2025 overview and analysis on *Antifungal Agents in Clinical and Preclinical Development* were two breaths of oxygen, legitimating our work and transforming it into an international public health priority.

## Figures and Tables

**Table 1:** Mechanisms of acquired resistance to the currently used antifungal agents.

Antifungals	Resistance Mechanisms	Documented Fungal Strains	References
Polyenes	Mutations in ergosterol biosynthesis genes, Accumulation of alternate sterols, Cell stress responses.	*Candida* spp., *C. neoformans*	[62]
Azoles	Mutations in drug target, Drug target overexpression, Efflux transporters overexpression, Cell stress responses.	*C. albicans*, *C. auris*, *A. fumigatus*	[62,89]
5-FC	Membrane cytosine permease mutation, Cytosine deaminase mutation.	*Cryptococcus* spp.	[50,53,90]
Echinocandins	Mutations in drug target, Cell stress responses (e.g., chitin synthase upregulation).	*Candida* spp., *Cryptococcus* spp., *Aspergillus* spp., *Saccharomyces cerevisiae*	[62,91,92]

## Data Availability

No new data were created or analyzed in this study. Data sharing is not applicable to this article.

## References

[1] Gow N.A.R., Johnson C., Berman J., Coste A.T., Cuomo C.A., Perlin D.S., Bicanic T., Harrison T.S., Wiederhold N., Bromley M. (2022). The importance of antimicrobial resistance in medical mycology. Nat. Commun..

[2] Hanafy D.M., Leaver D.J. (2025). Is a Fungal Apocalypse Inevitable or Just a Hallucination? An Overview of the Antifungal Armamentarium Used in the Fight against Pathogenic Fungi. ACS Med. Chem. Lett..

[3] Zhang Z., Bills G.F., An Z. (2023). Advances in the treatment of invasive fungal disease. PLoS Pathog..

[4] Denning D.W. (2024). Global incidence and mortality of severe fungal disease. Lancet Infect. Dis..

[5] Hibbett D., Nagy L.G., Nilsson R.H. (2025). Fungal diversity, evolution, and classification. Curr. Biol..

[6] Drexler M. (2010). How Infection Works. What You Need to Know About Infectious Disease.

[7] Pappas P.G., Chen S.C.-A., Donnelly J.P. (2021). The evidence supporting the revised EORTC/MSGERC definitions for invasive fungal infections. Clin. Infect. Dis..

[8] Bassetti M., Azoulay E., Kullberg B.-J., Ruhnke M., Shoham S., Vazquez J., Giacobbe D.R., Calandra T. (2021). EORTC/MSGERC definitions of invasive fungal diseases: Summary of activities of the intensive care unit working group. Clin. Infect. Dis..

[9] Donnelly J.P., Chen S.C., Kauffman C.A., Steinbach W.J., Baddley J.W., Verweij P.E., Clancy C.J., Wingard J.R., Lockhart S.R., Groll A.H. (2019). Revision and update of the consensus definitions of invasive fungal disease from the European organization for Research and Treatment of Cancer and the Mycoses Study Group Education and Research Consortium. Clin. Infect. Dis..

[10] Cornely O.A., Sprute R., Bassetti M., Chen S.C.-A., Groll A.H., Kurzai O., Lass-Flörl C., Ostrosky-Zeichner L., Rautemaa-Richardson R., Revathi G. (2025). Global guideline for the diagnosis and management of candidiasis: An initiative of the ECMM in cooperation with ISHAM and ASM. Lancet Infect. Dis..

[11] Brown G.D., Denning D.W., Levitz S.M. (2012). Tackling human fungal infections. Science.

[12] Köhler J.R., Hube B., Puccia R., Casadevall A., Perfect J.R. (2017). Fungi that infect humans. Microbiol. Spectr..

[13] Bongomin F., Gago S., Oladele R.O., Denning D.W. (2017). Global and multi-national prevalence of fungal diseases—Estimate precision. J. Fungi.

[14] Tsai C.-S., Lee S.S.-J., Chen W.-C., Tseng C.-H., Lee N.-Y., Chen P.-L., Li M.-C., Syue L.-S., Lo C.-L., Ko W.-C. (2023). COVID-19-associated candidiasis and the emerging concern of *Candida auris* infections. J. Microbiol. Immunol. Infect..

[15] Raut A., Huy N.T. (2021). Rising incidence of mucormycosis in patients with COVID-19: Another challenge for India against the second wave?. Lancet Respir. Med..

[16] Rouzé A., Lemaitre E., Martin-Loeches I., Povoa P., Diaz E., Nyga R., Torres A., Metzelard M., Du Cheyron D., Lambiotte F. (2022). Invasive pulmonary aspergillosis among intubated patients with SARS-CoV-2 or influenza pneumonia: A European multicenter comparative cohort study. Crit. Care.

[17] Kumar V., Huang J., Dong Y., Hao G.-F. (2024). Targeting Fks1 proteins for novel antifungal drug discovery. Trends Pharmacol. Sci..

[18] Fisher M.C., Denning D.W. (2023). The WHO fungal priority pathogens list as a game-changer. Nat. Rev. Microbiol..

[19] Firacative C. (2020). Invasive fungal disease in humans: Are we aware of the real impact?. Mem. Inst. Oswaldo Cruz.

[20] de Hoog S., Walsh T.J., Ahmed S.A., Alastruey-Izquierdo A., Alexander B.D., Arendrup M.C., Babady E., Bai F.-Y., Balada-Llasat J.-M., Borman A. (2023). A conceptual framework for nomenclatural stability and validity of medically important fungi: A proposed global consensus guideline for fungal name changes supported by ABP, ASM, CLSI, ECMM, ESCMID-EFISG, EUCAST-AFST, FDLC, IDSA, ISHAM, MMSA, and MSGERC. J. Clin. Microbiol..

[21] Rabaan A.A., Sulaiman T., Al-Ahmed S.H., Buhaliqah Z.A., Buhaliqah A.A., AlYuosof B., Alfaresi M., Al Fares M.A., Alwarthan S., Alkathlan M.S. (2023). Potential strategies to control the risk of antifungal resistance in humans: A comprehensive review. Antibiotics.

[22] Vanreppelen G., Wuyts J., Van Dijck P., Vandecruys P. (2023). Sources of Antifungal Drugs. J. Fungi.

[23] Chen F., Qasir D., Morris A.C. (2022). Invasive pulmonary aspergillosis in hospital and ventilator-associated pneumonias. Semin. Respir. Crit. Care Med..

[24] Rusu S., Lavis P., Domingues Salgado V., Van Craynest M.-P., Creteur J., Salmon I., Brasseur A., Remmelink M. (2021). Comparison of antemortem clinical diagnosis and post-mortem findings in intensive care unit patients. Virchows Arch..

[25] Bassetti M., Giacobbe D.R., Grecchi C., Rebuffi C., Zuccaro V., Scudeller L., The FUNDICU Investigators (2020). Performance of existing definitions and tests for the diagnosis of invasive aspergillosis in critically ill, adult patients: A systematic review with qualitative evidence synthesis. J. Infect..

[26] Candoni A., Farina F., Perruccio K., Di Blasi R., Criscuolo M., Cattaneo C., Delia M., Zannier M.E., Dragonetti G., Fanci R. (2020). Impact of invasive aspergillosis occurring during first induction therapy on outcome of acute myeloid leukaemia (SEIFEM-12B study). Mycoses.

[27] Fungal Disease Frequency|Gaffi—Global Action for Fungal Infections. https://gaffi.org/why/fungal-disease-frequency/.

[28] Limper A.H., Adenis A., Le T., Harrison T.S. (2017). Fungal infections in HIV/AIDS. Lancet Infect. Dis..

[29] Rajasingham R., Smith R.M., Park B.J., Jarvis J.N., Govender N.P., Chiller T.M., Denning D.W., Loyse A., Boulware D.R. (2017). Global burden of disease of HIV-associated cryptococcal meningitis: An updated analysis. Lancet Infect. Dis..

[30] Howard K.C., Dennis E.K., Watt D.S., Garneau-Tsodikova S. (2020). A comprehensive overview of the medicinal chemistry of antifungal drugs: Perspectives and promise. Chem. Soc. Rev..

[31] Ostrosky-Zeichner L., Casadevall A., Galgiani J.N., Odds F.C., Rex J.H. (2010). An insight into the antifungal pipeline: Selected new molecules and beyond. Nat. Rev. Drug Discov..

[32] Carmo A., Rocha M., Pereirinha P., Tomé R., Costa E. (2023). Antifungals: From pharmacokinetics to clinical practice. Antibiotics.

[33] Carolus H., Pierson S., Lagrou K., Van Dijck P. (2020). Amphotericin B and other polyenes-discovery, clinical use, mode of action and drug resistance. J. Fungi.

[34] Campoy S., Adrio J.L. (2017). Antifungals. Biochem. Pharmacol..

[35] Wang X., Mohammad I.S., Fan L., Zhao Z., Nurunnabi M., Sallam M.A., Wu J., Chen Z., Yin L., He W. (2021). Delivery strategies of amphotericin B for invasive fungal infections. Acta Pharm. Sin. B.

[36] Matsumori N., Sawada Y., Murata M. (2005). Mycosamine orientation of amphotericin B controlling interaction with ergosterol: Sterol-Dependent activity of conformation-restricted derivatives with an amino-carbonyl bridge. J. Am. Chem. Soc..

[37] Mehta D., Saini V., Bajaj A. (2023). Recent developments in membrane targeting antifungal agents to mitigate antifungal resistance. RSC Med. Chem..

[38] Puumala E., Fallah S., Robbins N., Cowen L.E. (2024). Advancements and challenges in antifungal therapeutic development. Clin. Microbiol. Rev..

[39] Cavassin F.B., Baú-Carneiro J.L., Vilas-Boas R.R., Queiroz-Telles F. (2021). Sixty years of Amphotericin B: An Overview of the Main Antifungal Agent Used to Treat Invasive Fungal Infections. Infect. Dis. Ther..

[40] Abraham D.J., Rotella D. (2021). Burger’s Medicinal Chemistry, Drug Discovery and Development.

[41] Hiemenz J.W., Walsh T.J. (1996). Lipid formulations of amphotericin B: Recent progress and future directions. Clin. Infect. Dis..

[42] Lee J.S.F., Cohen R.M., Khan R.A., Burry J., Casas E.C., Chung H.Y., Costa L.H., Ford N., Galvao D.L.N., Giron N. (2024). Paving the way for affordable and equitable liposomal amphotericin B access worldwide. Lancet Glob. Health.

[43] Cornely O.A., Alastruey-Izquierdo A., Arenz D., Chen S.C.A., Dannaoui E., Hochhegger B., Hoenigl M., Jensen H.E., Lagrou K., Lewis R.E. (2019). Global guideline for the diagnosis and management of mucormycosis: An initiative of the European Confederation of Medical Mycology in cooperation with the Mycoses Study Group Education and Research Consortium. Lancet Infect. Dis..

[44] Ullmann A.J., Aguado J.M., Arikan-Akdagli S., Denning D.W., Groll A.H., Lagrou K., Lass-Flörl C., Lewis R.E., Munoz P., Verweij P.E. (2018). Diagnosis and management of *Aspergillus* diseases: Executive summary of the 2017 ESCMID-ECMM-ERS guideline. Clin. Microbiol. Infect..

[45] World Health Organization (2020). Guidelines for Diagnosing and Managing Disseminated Histoplasmosis Among People Living with HIV.

[46] World Health Organization (2016). Guidelines for Diagnosing, Preventing and Managing Cryptococcal Disease Among Adults, Adolescents and Children Living with HIV.

[47] Heidelberger C., Chaudhuri N.K., Danneberg P., Mooren D., Griesbach L., Duschinsky R., Schnitzer R.J., Pleven E., Scheiner J. (1957). Fluorinated pyrimidines, a new class of tumour-inhibitory compounds. Nature.

[48] Tassel D., Madoff M.A. (1968). Treatment of *Candida* sepsis and *Cryptococcus* meningitis with 5-fluorocytosine. A new antifungal agent. JAMA.

[49] Vermes A., Guchelaar H.-J., Dankert J. (2000). Flucytosine: A review of its pharmacology, clinical indications, pharmacokinetics, toxicity and drug interactions. J. Antimicrob. Chemother..

[50] Groll A.H., Piscitelli S.C., Walsh T.J., August J.T., Anders M.W., Murad F., Coyle J.T. (1998). Clinical pharmacology of systemic antifungal agents: A comprehensive review of agents in clinical use, current investigational compounds, and putative targets for antifungal drug development. Advances in Pharmacology.

[51] Sigera L.S.M., Denning D.W. (2023). Flucytosine and its clinical usage. Ther. Adv. Infect. Dis..

[52] Delma F.Z., Al-Hatmi A.M.S., Brüggemann R.J.M., Melchers W.J.G., de Hoog S., Verweij P.E., Buil J.B. (2021). Molecular mechanisms of 5-fluorocytosine resistance in yeasts and filamentous fungi. J. Fungi.

[53] Gintjee T.J., Donnelley M.A., Thompson G.R. (2020). Aspiring antifungals: Review of current antifungal pipeline developments. J. Fungi.

[54] Houšť J., Spížek J., Havlíček V. (2020). Antifungal Drugs. Metabolites.

[55] AFECT (Association Française des Enseignants de Chimie Thérapeutique) (1996). Principaux Antifongiques et Antiparasitaires: Traité de Chimie Thérapeutique.

[56] Sheehan D.J., Hitchcock C.A., Sibley C.M. (1999). Current and emerging azole antifungal agents. Clin. Microbiol. Rev..

[57] European Medicines Agency (2014). Ketoconazole-Containing Medicines—Referral.

[58] Como J.A., Dismukes W.E. (1994). Oral azole drugs as systemic antifungal therapy. N. Engl. J. Med..

[59] Dahiya S., Sharma N., Punia A., Choudhary P., Gulia P., Parmar V.S., Chhillar A.K. (2022). Antimycotic drugs and their mechanisms of resistance to *Candida* Species. Curr. Drug Targets.

[60] Antimicrobial-Resistant Fungi|Fungal Diseases|CDC. https://www.cdc.gov/fungal/antimicrobial-resistant-fungi/index.html.

[61] Singh A., Singh K., Sharma A., Kaur K., Chadha R., Bedi P.M.S. (2023). Recent advances in antifungal drug development targeting lanosterol 14α-demethylase (CYP51): A comprehensive review with structural and molecular insights. Chem. Biol. Drug Des..

[62] Lee Y., Robbins N., Cowen L.E. (2023). Molecular mechanisms governing antifungal drug resistance. NPJ Antimicrob. Resist..

[63] Maertens J.A. (2004). History of the development of azole derivatives. Clin. Microbiol. Infect..

[64] Kim J.H., Suh J.W., Kim M.J. (2023). Evaluation of Fluconazole versus Echinocandins for Treatment of Candidemia Caused by Susceptible Common *Candida* Species: A Propensity Score Matching Analysis. J. Fungi.

[65] Cartau T., Chantepie S., Thuillier-Lecouf A., Langlois B., Bonhomme J. (2024). Epidemiology, Clinical, Radiological and Biological Characteristics, and Outcomes of Mucormycosis: A Retrospective Study at a French University Hospital. J. Fungi.

[66] Hüttel W. (2021). Echinocandins: Structural diversity, biosynthesis, and development of antimycotics. Appl. Microbiol. Biotechnol..

[67] Benz F., Knüsel F., Nüesch J., Treichler H., Voser W., Nyfeler R., Keller-Schierlein W. (1974). Stoffwechselprodukte von Mikroorganismen 143. Mitteilung. Echinocandin B, ein neuartiges Polypeptid-Antibioticum aus *Aspergillus nidulans* var. *echinulatus*: Isolierung und Bausteine. Helv. Chim. Acta.

[68] Balkovec J.M., Hughes D.L., Masurekar P.S., Sable C.A., Schwartz R.E., Singh S.B. (2014). Discovery and development of first in class antifungal caspofungin (CANCIDAS^®^)—A case study. Nat. Prod. Rep..

[69] Keller-Juslén C., Kuhn M., Loosli H.R., Petcher T.J., Weber H.P., von Wartburg A. (1976). Struktur des cyclopeptid-antibiotikums sl 7810 (Echinocandin B). Tetrahedron Lett..

[70] Kurtz M.B., Rex J.H. (2001). Glucan synthase inhibitors as antifungal agents. Advances in Protein Chemistry.

[71] Keating G.M., Jarvis B. (2001). Caspofungin. Drugs.

[72] Zaas A.K., Steinbach W.J. (2005). Micafungin: The US perspective. Expert. Rev. Anti Infect. Ther..

[73] Vazquez J.A., Sobel J.D. (2006). Anidulafungin: A novel echinocandin. Clin. Infect. Dis..

[74] Syed Y.Y. (2023). Rezafungin: First Approval. Drugs.

[75] Douglas C.M., D’Ippolito J.A., Shei G.J., Meinz M., Onishi J., Marrinan J.A., Li W., Abruzzo G.K., Flattery A., Bartizal K. (1997). Identification of the FKS1 gene of *Candida albicans* as the essential target of 1,3-beta-D-glucan synthase inhibitors. Antimicrob. Agents Chemother..

[76] Hu X., Yang P., Chai C., Liu J., Sun H., Wu Y., Zhang M., Zhang M., Liu X., Yu H. (2023). Structural and mechanistic insights into fungal β-1,3-glucan synthase FKS1. Nature.

[77] Hussain M.K., Ahmed S., Khan A., Siddiqui A.J., Khatoon S., Jahan S. (2023). Mucormycosis: A hidden mystery of fungal infection, possible diagnosis, treatment and development of new therapeutic agents. Eur. J. Med. Chem..

[78] Espinel-Ingroff A. (1998). Comparison of in vitro activities of the new triazole SCH56592 and the echinocandins MK-0991 (L-743,872) and LY303366 against opportunistic filamentous and dimorphic fungi and yeasts. J. Clin. Microbiol..

[79] Zhu B., Dong Y., Ma J., Chen M., Ruan S., Zhao W., Feng J. (2020). The synthesis and activity evaluation of N-acylated analogs of echinocandin B with improved solubility and lower toxicity. J. Pept. Sci..

[80] de la Torre P., Reboli A.C. (2014). Micafungin: An evidence-based review of its place in therapy. Core Evid..

[81] Bormann A.M., Morrison V.A. (2009). Review of the pharmacology and clinical studies of micafungin. Drug Des. Devel Ther..

[82] European Medicines Agency (2013). Mycamine|European Medicines Agency.

[83] World Health Organization (2025). Antifungal Agents in Clinical and Preclinical Development: Overview and Analysis.

[84] EUCAST: Breakpoints for Antifungals. https://www.eucast.org/astoffungi/clinicalbreakpointsforantifungals.

[85] Clinical & Laboratory Standards Institute Clinical & Laboratory Standards Institute: CLSI Guidelines. https://clsi.org/.

[86] Otto W.R., Arendrup M.C., Fisher B.T. (2023). A practical guide to Antifungal Susceptibility Testing. J. Pediatr. Infect. Dis. Soc..

[87] Arastehfar A., Gabaldón T., Garcia-Rubio R., Jenks J.D., Hoenigl M., Salzer H.J.F., Ilkit M., Lass-Flörl C., Perlin D.S. (2020). Drug-resistant fungi: An emerging challenge threatening our limited sntifungal armamentarium. Antibiotics.

[88] Fisher M.C., Alastruey-Izquierdo A., Berman J., Bicanic T., Bignell E.M., Bowyer P., Bromley M., Brüggemann R., Garber G., Cornely O.A. (2022). Tackling the emerging threat of antifungal resistance to human health. Nat. Rev. Microbiol..

[89] Czajka K.M., Venkataraman K., Brabant-Kirwan D., Santi S.A., Verschoor C., Appanna V.D., Singh R., Saunders D.P., Tharmalingam S. (2023). Molecular mechanisms associated with antifungal resistance in pathogenic *Candida* species. Cells.

[90] Yang D.-H., Khanal Lamichhane A., Kwon-Chung K.J., Chang Y.C. (2023). Factors influencing the nitrogen-source dependent flucytosine resistance in *Cryptococcus* species. mBio.

[91] Perlin D.S. (2015). Mechanisms of echinocandin antifungal drug resistance. Ann. N. Y. Acad. Sci..

[92] Lotfali E., Fattahi A., Sayyahfar S., Ghasemi R., Rabiei M.M., Fathi M., Vakili K., Deravi N., Soheili A., Toreyhi H. (2021). A review on molecular mechanisms of antifungal resistance in *Candida glabrata*: Update and recent advances. Microb. Drug Resist..

[93] Huang Y., Su Y., Chen X., Xiao M., Xu Y. (2024). Insight into virulence and mechanisms of Amphotericin B resistance in the *Candida haemulonii* complex. J. Fungi.

[94] Akinosoglou K., Rigopoulos E.A., Papageorgiou D., Schinas G., Polyzou E., Dimopoulou E., Gogos C., Dimopoulos G. (2024). Amphotericin B in the era of new antifungals: Where Will It Stand?. J. Fungi.

[95] De Francesco M.A. (2023). Drug-resistant *Aspergillus* spp.: A literature review of its resistance mechanisms and its prevalence in Europe. Pathogens.

[96] Ganesan P., Ganapathy D., Sekaran S., Murthykumar K., Sundramoorthy A.K., Pitchiah S., Shanmugam R. (2022). Molecular mechanisms of antifungal resistance in mucormycosis. BioMed. Res. Int..

[97] Lass-Flörl C., Steixner S. (2023). The changing epidemiology of fungal infections. Mol. Asp. Med..

[98] Pfaller M.A., Diekema D.J., Turnidge J.D., Castanheira M., Jones R.N. (2019). Twenty Years of the SENTRY Antifungal Surveillance Program: Results for *Candida* Species From 1997–2016. Open Forum Infect. Dis..

[99] Odoj K., Garlasco J., Pezzani M.D., Magnabosco C., Ortiz D., Manco F., Galia L., Foster S.K., Arieti F., Tacconelli E. (2024). Tracking candidemia trends and antifungal resistance patterns across Europe: An in-depth analysis of surveillance systems and surveillance studies. J. Fungi.

[100] Sanguinetti M., Posteraro P., Posteraro B. (2010). Echinocandin antifungal drug resistance in *Candida* species: A cause for concern?. Curr. Infect. Dis. Rep..

[101] Mroczyńska M., Brillowska-Dąbrowska A. (2020). Review on current status of echinocandins use. Antibiotics.

[102] Rasheed M., Battu A., Kaur R. (2020). Host-pathogen interaction in *Candida glabrata* infection: Current knowledge and implications for antifungal therapy. Expert. Rev. Anti Infect. Ther..

[103] Jospe-Kaufman M., Fridman M. (2025). Illuminating antifungal mode of action and resistance with fluorescent probes. Curr. Opin. Chem. Biol..

[104] Cass L., Murray A., Davis A., Woodward K., Albayaty M., Ito K., Strong P., Ayrton J., Brindley C., Prosser J. (2020). Safety and nonclinical and clinical pharmacokinetics of PC945, a novel inhaled triazole antifungal agent. Pharmacol. Res. Perspect..

[105] Hoenigl M., Sprute R., Egger M., Arastehfar A., Cornely O.A., Krause R., Lass-Flörl C., Prattes J., Spec A., Thompson G.R. (2021). The Antifungal Pipeline: Fosmanogepix, Ibrexafungerp, Olorofim, Opelconazole, and Rezafungin. Drugs.

[106] Pulmocide Ltd (2024). A Double-Blind, Randomized, Placebo-Controlled Study to Assess the Safety and Efficacy of Nebulized PC945 When Added to Systemic Antifungal Therapy for the Treatment of Refractory Invasive Pulmonary Aspergillosis (OPERA-T Study).

[107] Nishimoto A.T., Wiederhold N.P., Flowers S.A., Zhang Q., Kelly S.L., Morschhäuser J., Yates C.M., Hoekstra W.J., Schotzinger R.J., Garvey E.P. (2019). In vitro activities of the novel investigational tetrazoles VT-1161 and VT-1598 compared to the triazole antifungals against azole-resistant strains and clinical isolates of *Candida albicans*. Antimicrob. Agents Chemother..

[108] Wiederhold N.P., Najvar L.K., Garvey E.P., Brand S.R., Xu X., Ottinger E.A., Alimardanov A., Cradock J., Behnke M., Hoekstra W.J. (2018). The fungal Cyp51 inhibitor VT-1129 Is efficacious in an experimental model of Cryptococcal meningitis. Antimicrob. Agents Chemother..

[109] Wiederhold N.P., Shubitz L.F., Najvar L.K., Jaramillo R., Olivo M., Catano G., Trinh H.T., Yates C.M., Schotzinger R.J., Garvey E.P. (2018). The novel fungal Cyp51 inhibitor VT-1598 is efficacious in experimental models of central nervous system coccidioidomycosis caused by *Coccidioides posadasii* and *Coccidioides immitis*. Antimicrob. Agents Chemother..

[110] Oliver J.D., Sibley G.E.M., Beckmann N., Dobb K.S., Slater M.J., McEntee L., du Pré S., Livermore J., Bromley M.J., Wiederhold N.P. (2016). F901318 represents a novel class of antifungal drug that inhibits dihydroorotate dehydrogenase. Proc. Natl. Acad. Sci. USA.

[111] Wiederhold N.P. (2020). Review of the novel investigational antifungal olorofim. J. Fungi.

[112] F2G Biotech GmbH (2022). Phase IIb Study of F901318 as Treatment of Invasive Fungal Infections Due to Lomentospora Prolificans, Scedosporium spp., Aspergillus spp., and Other Resistant Fungi in Patients Lacking Suitable Alternative Treatment Options.

[113] F2G Biotech GmbH (2024). Phase III, Adjudicator-Blinded, Randomised Study to Evaluate Efficacy and Safety of Treatment with Olorofim Versus Treatment with AmBisome® Followed by Standard of Care in Patients with Invasive Fungal Disease Caused by Aspergillus Species.

[114] Shaw K.J., Ibrahim A.S. (2020). Fosmanogepix: A Review of the First-in-Class Broad Spectrum Agent for the Treatment of Invasive Fungal Infections. J. Fungi.

[115] Covel J., Soltow Q., Kapoor M., Moloney M., Webb P., Trzoss M., Sharp M., Shaw K., Trzoss M. (2019). The discovery of Manogepix/Fosmanogepix and other Gwt1 inhibitors for the treatment of invasive fungal infections. 2019 Medicinal Chemistry Reviews.

[116] Pfizer (2023). An Open-Label Study to Evaluate the Efficacy and Safety of APX001 in Patients with Candidemia and/or Invasive Candidiasis Caused by Candida Auris.

[117] Basilea Pharmaceutica (2024). An Interventional Efficacy and Safety Phase 3 Double-Blind 2-Arm Study to Investigate IV Followed by Oral Fosmanogepix Compared with IV Caspofungin Followed by Oral Fluconazole in Adult Participants with Candidemia and/or Invasive Candidiasis.

[118] Davis M.R., Donnelley M.A., Thompson G.R. (2020). Ibrexafungerp: A novel oral glucan synthase inhibitor. Med. Mycol..

[119] Lee A. (2021). Ibrexafungerp: First Approval. Drugs.

[120] Scynexis, Inc (2024). Open-Label Study to Evaluate the Efficacy and Safety of SCY-078 (Ibrexafungerp) in Patients with Fungal Diseases That Are Refractory to or Intolerant of Standard Antifungal Treatment (FURI).

[121] Stenland C.J., Lis L.G., Schendel F.J., Hahn N.J., Smart M.A., Miller A.L., von Keitz M.G., Gurvich V.J. (2013). A practical and scalable manufacturing process for an anti-fungal agent, Nikkomycin Z. Org. Process Res. Dev..

[122] Wu Y., Zhang M., Yang Y., Ding X., Yang P., Huang K., Hu X., Zhang M., Liu X., Yu H. (2022). Structures and mechanism of chitin synthase and its inhibition by antifungal drug Nikkomycin Z. Cell Discov..

[123] University of Arizona (2013). Phase I/II Evaluation of the Safety, Pharmacokinetics, and Preliminary Effectiveness of Nikkomycin Z in the Treatment of Patients with Uncomplicated Coccidioides Pneumonia.

[124] Zhou M., Liu L., Cong Z., Jiang W., Xiao X., Xie J., Luo Z., Chen S., Wu Y., Xue X. (2024). A dual-targeting antifungal is effective against multidrug-resistant human fungal pathogens. Nat. Microbiol..

[125] Shekhar-Guturja T., Gunaherath G.M.K.B., Wijeratne E.M.K., Lambert J.-P., Averette A.F., Lee S.C., Kim T., Bahn Y.-S., Tripodi F., Ammar R. (2016). Dual action antifungal small molecule modulates multidrug efflux and TOR signaling. Nat. Chem. Biol..

[126] Liu X., Li H., Qi G., Qian Y., Li B., Shi L., Liu B. (2024). Combating Fungal Infections and Resistance with a Dual-Mechanism Luminogen to Disrupt Membrane Integrity and Induce DNA Damage. J. Am. Chem. Soc..

[127] Yu S., He Y.-Q., Liu Y., Ji S., Wang Y., Sun B. (2024). Construction and Activity Evaluation of Novel Bifunctional Inhibitors and a COF Carrier Based on a Fungal Infection Microenvironment. J. Med. Chem..

[128] Cellini B., Pampalone G., Camaioni E., Pariano M., Catalano F., Zelante T., Dindo M., Macchioni L., Di Veroli A., Galarini R. (2023). Dual species sphingosine-1-phosphate lyase inhibitors to combine antifungal and anti-inflammatory activities in cystic fibrosis: A feasibility study. Sci. Rep..

[129] Yang Q., Xie J., Cai Y., Wang N., Wang Y., Zhang L., Li Y., Yu J., Li Y., Wang H. (2022). Efficacy and Safety of Combination Antifungals as Empirical, Preemptive, and Targeted Therapies for Invasive Fungal Infections in Intensive-Care Units. Infect. Drug Resist..

[130] Toepfer S., Keniya M.V., Lackner M., Monk B.C. (2024). Azole Combinations and Multi-Targeting Drugs That Synergistically Inhibit *Candidozyma auris*. J. Fungi.

